# Development of a Rapid Agglutination Latex Test for Diagnosis of Enteropathogenic and Enterohemorrhagic *Escherichia coli* Infection in Developing World: Defining the Biomarker, Antibody and Method

**DOI:** 10.1371/journal.pntd.0003150

**Published:** 2014-09-25

**Authors:** Letícia B. Rocha, Anna R. R. Santos, Danielle D. Munhoz, Lucas T. A. Cardoso, Daniela E. Luz, Fernanda B. Andrade, Denise S. P. Q. Horton, Waldir P. Elias, Roxane M. F. Piazza

**Affiliations:** Laboratório de Bacteriologia, Instituto Butantan, São Paulo, São Paulo, Brazil; Institut Pasteur, France

## Abstract

**Background:**

Enteropathogenic and enterohemorrhagic *Escherichia coli* (EPEC/EHEC) are human intestinal pathogens responsible for diarrhea in both developing and industrialized countries. In research laboratories, EPEC and EHEC are defined on the basis of their pathogenic features; nevertheless, their identification in routine laboratories is expensive and laborious. Therefore, the aim of the present work was to develop a rapid and simple assay for EPEC/EHEC detection. Accordingly, the EPEC/EHEC-secreted proteins EspA and EspB were chosen as target antigens.

**Methodology:**

First, we investigated the ideal conditions for EspA/EspB production/secretion by ELISA in a collection of EPEC/EHEC strains after cultivating bacterial isolates in Dulbecco’s modified Eagle’s medium (DMEM) or DMEM containing 1% tryptone or HEp-2 cells-preconditioned DMEM, employing either anti-EspA/anti-EspB polyclonal or monoclonal antibodies developed and characterized herein. Subsequently, a rapid agglutination latex test (RALT) was developed and tested with the same collection of bacterial isolates.

**Principal findings:**

EspB was defined as a biomarker and its corresponding monoclonal antibody as the tool for EPEC/EHEC diagnosis; the production of EspB was better in DMEM medium. RALT assay has the sensitivity and specificity required for high-impact diagnosis of neglected diseases in the developing world.

**Conclusion:**

RALT assay described herein can be considered an alternative assay for diarrhea diagnosis in low-income countries since it achieved 97% sensitivity, 98% specificity and 97% efficiency.

## Author Summary

A rapid and low-cost diagnosis for EPEC/EHEC infections is extremely required considering their global prevalence, the severity of the diseases associated with them, and the fact that the use of antibiotics to treat EHEC infections can be harmful. For EHEC, the detection of Stx toxins has already been developed, but for EPEC, an internationally recognized standard diagnostic test is lacking. Thus, the approach for their rapid detection in this study was the use of the secreted proteins EspA and/or EspB, since they are the major secreted proteins in both pathogens. EspB was defined as a biomarker and its corresponding monoclonal antibody as the tool for EPEC/EHEC diagnosis using a latex agglutination assay, which can be employed in less equipped laboratories in developing countries.

## Introduction

Annually, nearly five million cases of diarrhea are reported around the world leading to 800 thousand deaths per year in under-fives [1], [2], and *Escherichia coli* is the etiological agent responsible for most of them [3]. The *E. coli* isolates associated with diarrhea are classified into pathotypes on the basis of specific virulence factors, pathogenesis or clinical manifestation [4]. Among them, enteropathogenic *E. coli* (EPEC) and enterohemorrhagic *E. coli* (EHEC) continue to represent a threat to human health worldwide [5].

Both pathotypes can induce the attaching and effacing (A/E) lesion on the intestinal mucosa, characterized by intimate bacterial adhesion, destruction of microvilli, and accumulation of polymerized actin in pedestals beneath intimately attached bacteria [6]. The A/E lesion formation is caused by effector proteins that are secreted into the enterocytes by the type III secretion system [4]. All genes necessary for the A/E lesion formation are located in a pathogenicity island called locus of enterocyte effacement (LEE). After the establishment of initial contact via EspA containing filaments, two further effector proteins, EspB and EspD, are translocated into the host cell membrane where they form a pore structure [7], [8], which allows the translocation of effector proteins. The delivery of the translocated intimin receptor (Tir) into the host cell membrane is followed by dissolution of EspA filaments and intimate bacterial attachment via binding of Tir to the bacterial adhesin intimin [9], [10].

EHEC but not EPEC produces the Shiga toxins, which are associated with the development of severe complications of infection, namely hemorrhagic colitis (HC) and the hemolytic uremic syndrome (HUS) [11]. Moreover, some EPEC strains may carry a large plasmid known as the EPEC adherence factor plasmid (pEAF) [12], [13], which encodes the bundle-forming pilus (BFP) [14], [15]. Since pEAF is not present and BFP is not produced by all isolates, this pathotype has been divided in the subgroups typical EPEC (tEPEC) and atypical EPEC (aEPEC), where BFP is produced only by tEPEC [14], [16]–[18].

Epidemiologically, EHEC is more common as a food or water-borne pathogen in industrialized countries, and EPEC remains a significant cause of diarrhea in low-income countries, responsible for high rates of infant morbidity and mortality [15], [19], [20], but it is worth to mention that aEPEC has been now considered an emerging pathogen in both industrialized and developing countries [21]–[27].

EPEC and EHEC have been defined on the basis of their pathogenic properties; however, this detection in routine laboratories is expensive and laborious for developing countries. Therefore, in these settings they are defined only with a serogroup agglutination-based test [28]. As LEE-encoded virulence factors are common to EPEC and EHEC strains, intimin has been considered the first target for diagnosis [29], mainly its conserved region (Int_388–667_) [30], [31]. Essentially, intimin detection in EPEC and EHEC isolates is appropriate by immunofluorescence and/or immunoblotting, i.e., after bacterial permeabilization, allowing anti-intimin antibody accessibility [32]–[34].

Alternative targets for EPEC/EHEC diagnosis are the LEE-secreted proteins, including EspA and EspB. For production and delivery of EspA and EspB, special culture conditions are required [7], [9], [35]–[38]. Only a few developed antibodies against EspA or EspB have been used either in the characterization of EPEC or EHEC [7], [39], [40], and only anti-EspB polyclonal antibodies have been evaluated for diagnosis [41], [42]. Therefore the goal of the present work was to develop a rapid and simple assay for EPEC/EHEC detection, especially for EPEC, a pathotype that lacks an internationally recognized standard diagnostic test [43]. Accordingly, we first investigated the ideal conditions for EspA/EspB production/secretion in a collection of EPEC/EHEC isolates, employing either anti-EspA/anti-EspB polyclonal or monoclonal antibodies developed and characterized herein. Subsequently, we defined EspB as a target antigen and EspB monoclonal antibodies as a tool for the rapid agglutination latex test (RALT) to be considered an alternative assay for diarrhea diagnosis in developing countries.

## Methods

### Bacterial isolates

We analyzed in this study a collection of 71 aEPEC [17], 31 tEPEC [18], [32] and 23 EHEC [44], belonging to different serotypes characterized as LEE-positive isolates. We also included for ELISA cut-off definition and specificity of the RALT, 20 LEE-negative diarrheagenic *E. coli* (DEC/LEE^−^), 20 fecal *E. coli* negative for DEC virulence factors (NVF *E. coli*) isolates and 20 Enterobacteriaceae isolates (*Aeromonas hydrophila*, *Edwardsiella tarda*, *Enterobacter cloacae*, *Enterococcus faecalis*, *Klebsiella pneumoniae*, *Morganella morganii, Pseudomonas aeruginosa*, *Proteus mirabilis*, *Providencia spp*., *Salmonella spp.*, *Serratia marcescens*, *Shigella boydii* and *Shigella flexneri*) from our laboratory collection. The prototype tEPEC E2348/69 [45] was included in the assays as a positive control for EspA/EspB-producing strain.

### Ethics statement

These experiments were conducted in agreement with the Ethical Principles in Animal Research, adopted by the Brazilian College of Animal Experimentation, and they were approved by the Ethical Committee for Animal Research of Butantan Institute (Protocol 469/08).

EspA and EspB antibodies: development and characterization

EspA and EspB recombinant proteins were obtained from *E. coli* BL21 clones containing the pET28a-EspA or pET28a-EspB plasmid. Protein induction, production and purification were done as described elsewhere [7]. These proteins were employed for raising the rabbit polyclonal (PAb) [31] and the monoclonal (MAb) antibodies [44], [46].

### Detection of secreted proteins EspA and EspB

Bacterial isolates were cultivated in Luria Bertani (LB) broth at 37°C for 18 h. Each culture was then inoculated at a 1∶50 dilution at 37°C for 6 h (250 rpm) into Dulbecco’s modified Eagle’s medium (DMEM), DMEM containing 1% tryptone (DMEM-T) or preconditioned DMEM (DMEM-PC), which was prepared by incubation of DMEM without antibiotics or fetal bovine serum with monolayers of HEp-2 for 24–48 h. The supernatant referred to as “preconditioned medium” was collected, adjusted to pH 7.4, and filtered through a 0.2 mm membrane [47].

After growth of the bacteria, the cultures were centrifuged at 13,000×*g* for 10 min and the supernatants were stored at 4°C for 16–18 h. A 100-µL aliquot of supernatants was used to coat the microplates in indirect ELISA assays. The microplates (MaxiSorp microplates, Nunc, Rochester, NY, USA) were then kept at 37°C for 2 h. After blocking with 1% bovine serum albumin (BSA) at 37°C for 30 min, the microplates were incubated with anti-EspA MAb (5 µg/mL) or MAb anti-EspB (10 µg/mL) or with 30 µg/mL anti-EspA PAb or anti-EspB PAb at 37°C for 1 h. Antigen-antibody binding was detected by the addition of either peroxidase-conjugated goat anti-mouse IgG (1∶5,000) or peroxidase-conjugated goat anti-rabbit IgG (1∶5,000) and OPD (0.5 mg/mL) and H_2_O_2_ as enzyme substrates. The peroxidase reaction was stopped by the addition of 1 N HCl. The absorbance was measured at 492 nm in a Multiskan EX ELISA reader (Labsystems, Milford, MA, USA). The absorbance values from duplicates of three independent experiments from LEE-positive and LEE-negative isolates after reaction with anti-EspA or anti-EspB antibodies were analyzed by GraphPrism 5.01, using Student’s *t*-test and two-away ANOVA. The differences were considered statistically significant when p≤0.05. The receiver operating characteristic (ROC) curve was employed for determining the cut-off value using the ELISA data, considering the highest sensitivity and specificity.

### Rapid agglutination latex test (RALT)

Prior to testing the isolates by rapid agglutination latex test (RALT), the beads were coupled with anti-EspB MAb. Briefly, beads in a 2.5% aqueous suspension (1 µm diameter – Polyscience, Warrington, PA, USA) were washed three times with PBS and incubated with 8% glutaraldehyde in PBS at room temperature for 4 h. Next, 200 µg anti-EspB MAb were added and the mixture incubated at room temperature for 16–18 h for coupling, followed by further incubation in the presence of 0.2 M ethanolamine and BSA. Both incubations were with gentle mixing at room temperature for 30 min. Between incubations, the coated beads were washed and centrifuged (7,200×*g*) for 6 min. After the last washing procedure, the pellet was ressuspended in the storage buffer (Polyscience, Warrington, PA, USA) and kept at 4°C for 7 days. For RALT, bacterial lysate was prepared using 20 mg of isolates grown on DMEM-agar at 37°C for 16–18 h and suspended in 80 µL of lysis buffer [Bacterial Protein Extraction Reagent (B-PER), Thermo Scientific, Rockford, IL, USA], followed by incubation at room temperature for 15 min. The assay was performed on a slide glass using 20 µL of bacterial lysate and 20 µL of latex beads coupled to anti-EspB MAb, and checking for agglutination after 5 min of gentle mixing.

## Results

### Characteristics of anti-EspA and anti-EspB antibodies

EspA and EspB are noteworthy antigens, demonstrated by the anti-EspA and anti-EspB polyclonal antibodies IgG titers (1∶10,240 and 1∶40,960; respectively) and detection limit of 78 and 156 ng/mL, respectively. Secretory hybridomas of antibodies against EspA and EspB were obtained and subcloned by limiting dilution. Anti-EspA and anti-EspB MAbs produced by the selected clones (3C12 and 4D9, respectively), were classified as IgG2a and showed a dissociation constant of 1.66×10^−10^ and 2×10^−9^ M, with detection limit of 19 and 17 ng/mL, respectively.

### Production of secreted EspA and EspB proteins

**Figure 1:**
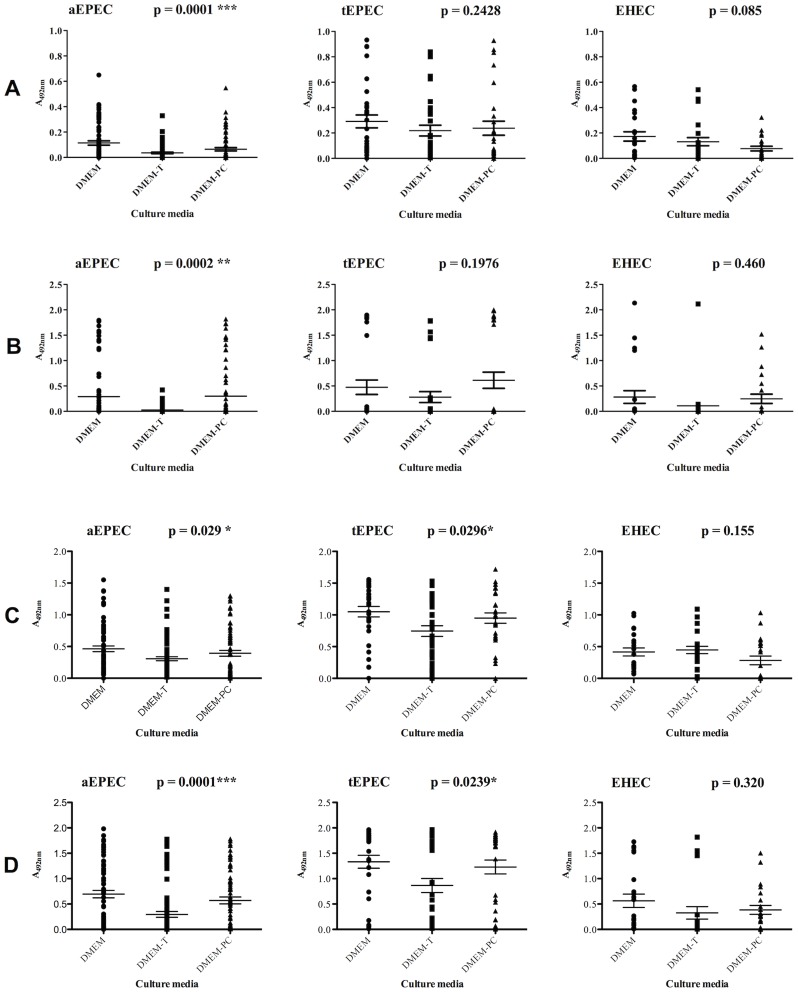
EspA and EspB production in different culture media. Atypical EPEC (aEPEC), typical EPEC (tEPEC) and EHEC isolates were cultivated in DMEM or DMEM-T or DMEM-PC. The supernatants were tested by indirect ELISA for EspA detection using anti-EspA IgG-enriched fraction (30 µg/mL) (**A**) and anti-EspA MAb (5 µg/mL) (**B**) and for EspB detection using anti-EspB IgG-enriched fraction (30 µg/mL) (**C**) and anti-EspB MAb (10 µg/mL) (**D**). The optical densities obtained for the isolates reacted with anti-EspA or anti-EspB polyclonal or monoclonal antibodies were analyzed by GraphPrism 5.01, using Student’s *t* test and two-away ANOVA. The differences were considered statistically significant when p≤0.05.

**Figure 2:**
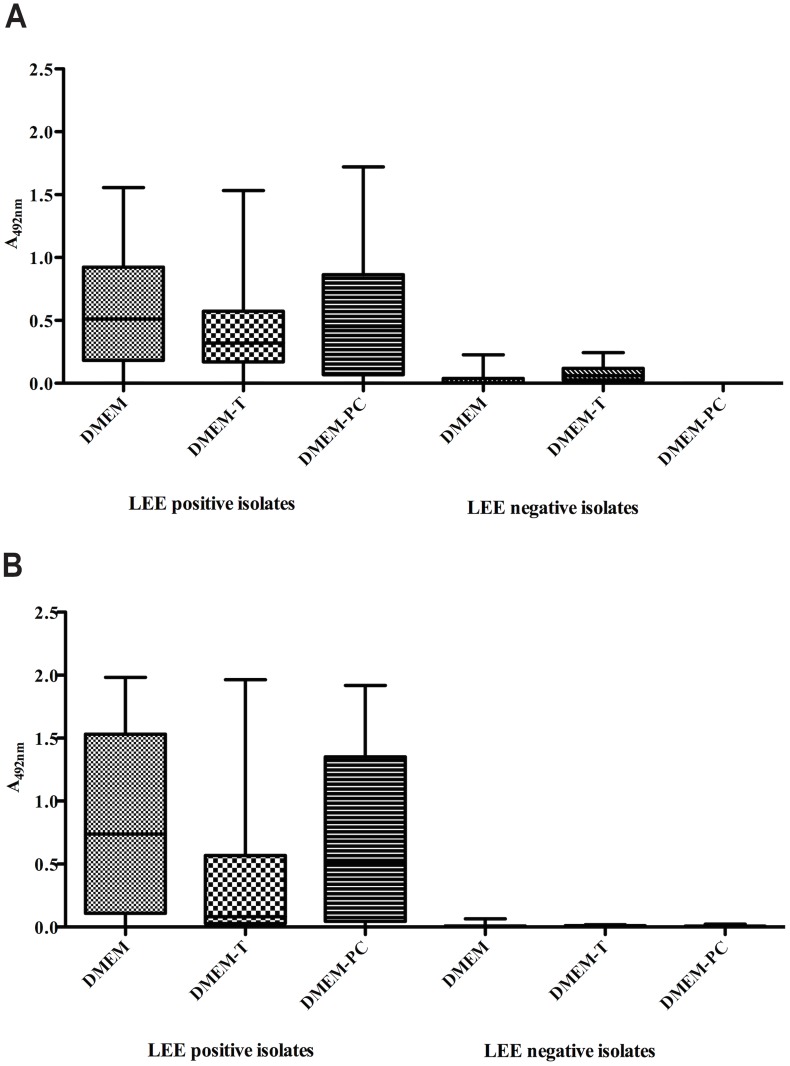
EspB production in different culture media. LEE-positive and LEE-negative isolates were cultivated in DMEM or DMEM-T or DMEM-PC. The supernatants were tested by indirect ELISA for EspB detection using anti-EspB IgG-enriched fraction (30 µg/mL) (**A**) and anti-EspB MAb (10 µg/mL) (**B**). The mean optical densities for LEE-negative and LEE-positive isolates were determined. The cut-off obtained by the ROC curve for anti-EspB MAb was 0.027 for DMEM and 0.0145 for DMEM-T and DMEM-PC. For anti-EspB PAb was 0.152 for DMEM, 0.135 for DMEM-T and 0.001 for DMEM-PC.

The reactivity of all antibodies, as well as the efficiency of different culture media (DMEM, DMEM-T and DMEM-PC) were determined in the collection of tEPEC, aEPEC and EHEC isolates by indirect ELISA. Using either anti-EspA PAb or anti-EspA MAb, the production of EspA by tEPEC and EHEC isolates was the same regardless the culture medium (**Figure 1** **A and B**). Considering either anti-EspB PAb or MAb, production of EspB by EHEC isolates was also medium independent. On the other hand, when LEE-positive isolates were evaluated as a group, the production of EspB was higher in DMEM compared to DMEM-T (p<0.0001) or to DMEM-PC (p = 0.003), and no difference was observed between DMEM-PC and DMEM-T (p = 0.129) (**Figure 2**).

Therefore, comparing the production of EspB in 125 LEE-positive and 60 LEE-negative isolates using both anti-EspB antibodies, we observed by ROC curves that regardless the medium employed the sensitivity (Se) and specificity (Sp) were higher with MAb (Se = 90.4%, confidence intervals of 83.8 to 96.4% and Sp = 96.4%, confidence intervals of 87.7 to 99.6%) (**Figure 2B**) than PAb (Se = 82.4%, confidence intervals of 74.6 to 88.6% and Sp = 92.9%, confidence intervals of 82.7 to 98%) (**Figure 2A**). And the ELISA cut-off value was lower for MAb than for PAb (0.027 and 0.152, respectively).

### Rapid agglutination latex test (RALT) using EspB as biomarker

**Figure 3:**
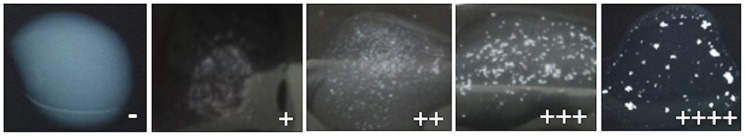
Typical of agglutination latex assay: negative and a semi-quantitative positive (from + to ++++) agglutination pattern with anti-EspB MAb coated beads. The test control with lysis buffer (B-PER) showed the same pattern as LEE-negative isolates.

For RALT, bacterial isolates were grown on DMEM-agar and the test was done using latex sensitized with anti-EspB MAb. Figure 3 presents typical negative and semi-quantitative positive reactions. From the total of the positive reactions by RALT, + correspond to 44.8%; ++ to 26.4%; +++ to 11.2% and ++++ to 14.4% of the isolates. By this assay only four LEE-positive isolates (one aEPEC and three tEPEC) did not react with anti-EspB MAb and one false positive occurred (*Proteus mirabilis*) (**Table 1**). Considering the LEE-positive and -negative isolates, the test exhibited 97% sensitivity, 98% specificity and 97% efficiency.

**Table 1:** Rapid agglutination latex test reactivity (%) with bacterial isolates.

Pathotype or group	No. of bacterial isolates	*espB* gene	Reactivity (%)	Total
aEPEC	71	**+**	98.6	70/71
tEPEC	31	**+**	90.3	28/31
EHEC	23	**+**	100	23/23
DEC/LEE^−^	20	**-**	0	0/20
NVF *E. coli*	20	**-**	0	0/20
Enterobacteriaceae (other than *E. coli*).	20	**-**	5	1^a^/20

tEPEC (typical enteropathogenic *E. coli*); aEPEC (atypical enteropathogenic *E. coli*); EHEC (enterohemorrhagic *E. coli*); DEC/LEE^−^ (LEE-negative diarrheagenic *E. coli*); NVF *E. coli* (fecal *E. coli* negative for DEC virulence factors).

a *Proteus mirabilis*.

## Discussion

A fast and inexpensive diagnosis for EPEC/EHEC infections is highly required considering their global prevalence, the severity of the diseases associated with them, and the fact that the use of antibiotics to treat EHEC infections can be harmful. One appropriate approach for their rapid detection may utilize the secreted proteins EspA and/or EspB, since the *espA* and e*spB* genes are present in LEE positive isolates and they are the major secreted proteins by both pathogens [4]. Thus, the aim of the present study was to develop and define sensitivity and specificity of EspA and EspB antibodies, determine the ideal target antigen, and design a simple and rapid test for the diagnosis of both emerging pathogens worldwide.

Production and secretion of virulence factors in pathogenic bacteria are tightly and coordinately regulated. Growth phase and environmental conditions characteristic of the host, including temperature and partial O_2_ pressure, are the stimulus for virulence factor expression in various gram-negative pathogens [48], [49]. Additionally, in our experience, the production of virulence factors is a critical point for diarrheagenic *E. coli* diagnosis [50]–[52].

Thus, initially, one group of isolates (including tEPEC, aEPEC and EHEC) was cultivated in different media: LB broth, DMEM, *E. coli* broth and Minimum medium. Besides, other culture conditions were tested, including pH (7.2 and 5.5), CO_2_ presence, and growth time period (6, 18 and 24 h). Our results showed that in general DMEM favored the production of secreted proteins after 6-h growth culture, but with individual variation (data not shown). Some reports describe that the use of preconditioned DMEM (DMEM-PC) provides signals from epithelial cells affecting virulence factors expression [47]. Also the secretion of plasmid-encoded toxin (Pet) by enteroaggregative *E. coli* is dependent on the addition of tryptone to DMEM (DMEM-T) [53]. Considering this, the bacterial isolates from our collection were cultivated in DMEM, DMEM-T and DMEM-PC, but EspB production and secretion was enhanced when bacterial isolates were cultivated in DMEM without enrichment.

Another important point of the present work was the evaluation of the four antibodies raised. We expected that EspA would be a biomarker for diagnosis and anti-EspA antibodies a detecting tool, since this protein is the major component of a transiently expressed surface organelle, which forms a direct link between the bacterium and the host cell [7]. However, our data pointed out EspB as the target antigen, and MAb anti-EspB the best antibody for defining LEE-positive isolates. Nakasone et al. [42], [54] also defined EspB as the target antigen for identifying LEE-positive strains. In fact, EspA filaments exhibit antigenic polymorphisms [55].

The indirect ELISA using anti-EspB MAb showed 90.4% and 96.4%, sensitivity and specificity, respectively, indicating its possible use in routine diagnostic laboratories. However, this methodology requires specific laboratory instrumentation, making it difficult to be performed in low-income country settings. Therefore, we standardized here a rapid agglutination test using latex beads coated with anti-EspB MAb (RALT), which has the sensitivity and specificity required for high impact diagnosis of neglected diseases in the developing world [56]. Two other assays have been described for LEE-positive isolates based on EspB detection; the 16–18 h reversed passive latex agglutination test (RPLA) [41] and a 10 min immunochromatographic test (IC) [42]. Although more time consuming, the RPLA test was more sensitive than the IC test [42].

Serotyping-based diagnosis is the only methodology available in limited-resources settings, employing either commercial or in-house antisera [28]. The standardized RALT for detection of EPEC and EHEC will have a remarkable impact in the diagnosis of these pathotypes, demonstrated by 97% sensitivity, 98% specificity and 97% efficiency in EspB detection. Also, no cross-reaction was observed with other DEC pathotypes and *E. coli* negative for DEC virulence factors. Among the enterobacteria species only one *Proteus mirabilis* was recognized by MAb anti-EspB. However, *P mirabilis* can be easily differentiated from EPEC/EHEC by biochemical methods employed for species identification [57], a step necessary prior to the performance of our RALT. Thus the established agglutination latex in the present study is a simple, rapid (5 min) and easy to perform test, which can be employed in less equipped laboratories in low-income countries.

## Supporting Information

Checklist S1 STARD checklist.
(DOC)
